# Dispersed Sensing Networks in Nano-Engineered Polymer Composites: *From Static Strain Measurement to Ultrasonic Wave Acquisition*

**DOI:** 10.3390/s18051398

**Published:** 2018-05-02

**Authors:** Yehai Li, Kai Wang, Zhongqing Su

**Affiliations:** Department of Mechanical Engineering, The Hong Kong Polytechnic University, Hung Hom, Kowloon, Hong Kong SAR 999077, China; yehai.li@connect.polyu.hk (Y.L.); kai-qf.wang@connect.polyu.hk (K.W.)

**Keywords:** nanocomposite sensor, self-sensing, ultrasonic guided waves, structural health monitoring, graphene nanoparticle

## Abstract

Self-sensing capability of composite materials has been the core of intensive research over the years and particularly boosted up by the recent quantum leap in nanotechnology. The capacity of most existing self-sensing approaches is restricted to static strains or low-frequency structural vibration. In this study, a new breed of functionalized epoxy-based composites is developed and fabricated, with a graphene nanoparticle-enriched, dispersed sensing network, whereby to self-perceive broadband elastic disturbance from static strains, through low-frequency vibration to guided waves in an ultrasonic regime. Owing to the dispersed and networked sensing capability, signals can be captured at any desired part of the composites. Experimental validation has demonstrated that the functionalized composites can self-sense strains, outperforming conventional metal foil strain sensors with a significantly enhanced gauge factor and a much broader response bandwidth. Precise and fast self-response of the composites to broadband ultrasonic signals (up to 440 kHz) has revealed that the composite structure itself can serve as ultrasound sensors, comparable to piezoceramic sensors in performance, whereas avoiding the use of bulky cables and wires as used in a piezoceramic sensor network. This study has spotlighted promising potentials of the developed approach to functionalize conventional composites with a self-sensing capability of high-sensitivity yet minimized intrusion to original structures.

## 1. Introduction

Nanoparticles which are dispersed in polymer matrices as reinforcement elements have now found their superb niches to functionalize conventional composites with new capabilities, as typified by electro-static discharge [1], electromagnetic interference shield [2], gas leakage sensing [3], UV-absorbing [4], flame-retardant coating [5] and damage detection [6,7], to name a few. In particular, the self-sensing using embedded nanoparticles has gained prominence in recent development of composites, to accommodate the increased desire from industry to acquire structural parameters and ambient information, not at the cost of introducing excessive weight and volume penalty to original composites due to the use of additional sensing systems—a paramount factor to be particularly considered for developing and implementing structural health monitoring (SHM) approaches in aerospace applications.

Conventionally, the self-sensing of composites can be achieved via the instinct electrical conductivity of carbon fibres in carbon fibre-reinforced polymers (CFRPs) [8] or via the introduction of optic fibres embedded in matrices [9]. However, as envisaged, the use of continuous carbon fibres as sensing elements through electrical resistance measurements shows fairly limited sensing capacity. In most demonstrated applications, it can respond only to the changes in electrical resistance induced by severe damage such as fibre breakage or delamination, and fail to perceive embryonic damage such as matrix cracking. On the other hand, to embed a fragile optical fibre, whose diameter is usually several times larger than that of a carbon fibre, usually incurs stress concentration and degrades local mechanical integrity of the composites.

Driven by the recent advances and technical breakthroughs in emerging nanotechnology, conductive nanoparticles in a diversity of modalities have become appealing nanofiller candidates to compete with conventional sensing elements in composites. These conductive nanoparticles can be dispersed in matrices to form conductive networks for strain sensing or damage detection [10,11,12,13]. The nanoscale of nanoparticles allows them to be well dispersed among continuous fibres in matrices. Rather than degrading the local mechanical strength of host composites, this even toughens the original materials if nanoparticles dispersed properly. There is a rich body of literature focusing on the recent development of such a research area, as well surveyed elsewhere [10,14,15]. Representatively, Thostenson et al. [16,17] demonstrated stable and sensitive acquisition of quasi-static and cyclic tensile strains by measuring the changes in electrical resistance of fibre composites with dispersed carbon nanotubes. In that study, the onset and progressing of damage that led to breakage of carbon nanotubes-formed conductive networks were linked to the increase of measured electrical resistance, whereby the severity of damage was estimated. In a similar vein, Nofar et al. [18] monitored damage progressing in a nanotube-fibre-epoxy composite laminate during a fatigue test, by measuring the changes in electrical resistance in the conductive network formed by a carbon nanotube-based network in the laminate. The approaches in this kind, relying on capturing a holistic change in electrical resistance (due to damage) of the entire sample show effectiveness in sensing occurrence of damage only, but they normally fail to render quantitative information (e.g., location, severity, type of damage). That is because the damage—local material degradation—in principle cannot be quantified using global parameters (e.g., electrical resistance). To overcome such a deficiency, Tallman et al. [19,20,21,22] and Naghashpour et al. [23], respectively, developed measurement approaches based on electrical impedance and resistance tomography, in which multiple electrodes were either painted across the surface of the samples [23,24], or dispersed along the boundaries of samples [19,25], to mesh the measurement area into sub-region. Each sensor was expected to capture local signals in the sub-region where it stayed. With information on local material degradation, these approaches are able to locate and quantify damage such as impacted holes [20,23,24,25,26].

However, the capacity of such a sensing philosophy is usually restricted to static strains or low-frequency and large magnitude structural vibration signals. This has created a vast barrier that prevents the functionalized composites with self-sensing from being extended to high-precision SHM, in which high-frequency probing signals in an ultrasonic regime are preferred, in order to make use of their superb sensitivity (at higher frequency) to damage of small dimensions. Of particular interest is the ultrasonic guided waves (UGWs) in the order of hundred kilohertz which demonstrate reasonable compromise among detection precision, fast propagation and penetration through composite thickness. When a probing UGW traverses a composite structure, rich information can be documented in captured signals, on which basis the damage in the composites along UGW propagation path, if any, can be scrutinized in a quantitative manner. But, it is noteworthy that UGW signals usually feature ultra-low magnitude at a microstrain degree, making it a daunting task to capture them using conventional nanoparticle-driven composites.

In conclusion, existing self-sensing of composites either cannot extract sufficient and effective information on damage or entails a dense grid of electrodes to be installed on the monitored area. Most importantly, they are unwieldy to respond, effectively and accurately, to UGWs propagating in the composites, let alone use of UGW signals for SHM of composites. Motivated by such recognition, in this study, we develop a new breed of nano-engineered polymer composites with dispersed and networked sensing capability. It can be responsive to broadband elastic disturbances from static strains, through low-frequency vibration to UGWs up to hundred kilohertz. To such an end, carbon-based nanoparticles are dispersed in polymer matrix and networked to form a self-sensing system. This dispersed nanoparticle-networked sensing system—denoted by Nano-NSS hereinafter—enables UGW acquisition at any desired part of the structure under monitoring. With ignorable weight penalty, a Nano-NSS can identify damage in a composites structure in a quantitative manner, and consequently evaluate its health status. Based on comparison among various nanoparticle candidates, a graphene nanoparticle-networked self-sensing system (gNano-NSS) is developed. The premise of gNano-NSS-based SHM lies in the quantum tunneling effect present in a built-in conductive network in the composites that is formed by two-dimensional graphene nanoparticles evenly dispersed in polymers. The sensing capability of gNano-NSS is tested by measuring quasi-static strains (high-level strains) in tensile tests, low-frequency dynamic signals (medium-level strains) in vibration tests, and high-frequency UGW signals (low-level strains) in ultrasound tests. Conventional metal foil strain gauges and piezoceramic wafers are used for calibration and comparison.

## 2. Material Preparation

### 2.1. Principle of Nanoparticle-Networked Self-Sensing System (Nano-NSS)

**Figure 1:**
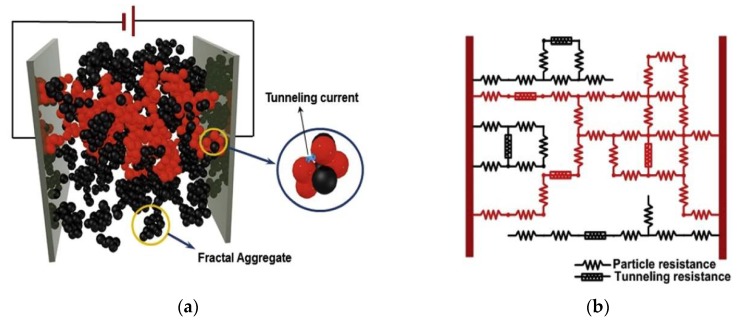
(**a**) Schematic of nano-NSS; (**b**) Equivalent resistor network (formed by directly-contacted nanoparticles and indirectly-contacted nanoparticles via the quantum tunneling effect).

In principle, with an increase in the number of nanoparticles in insulative polymer matrix, the holistic conductivity of a nanoparticle-enriched composites structure enhances progressively; and the *percolation threshold* represents such a critical volume fraction of nanoparticles in the matrix beyond which the quantum tunneling effect can be triggered among neighboring non-contacting conductive nanoparticles. The tunneling effect stimulates the formation of a full conductive network macroscopically manifested by the composite. Making use of the tunneling effect, the Nano-NSS achieves its sensing capability via calibrating the changes in nano-structure of the composites under strains that are induced as a result of UGW propagation. The mechanism behind such a sensing capability lies in a hybrid nature of the quantum tunneling effect and breakage of electric paths as a result of applied strains. The Nano-NSS responds to dynamic strains of the host structure between a pair of electrodes, and can perceive UGWs consisting of multiple wave modes propagating omnidirectionally when UGWs traverse Nano-NSS. The piezoresistivity manifested by Nano-NSS originates from induced changes in electrical resistance of the nanoparticles-formed conductive network that is dispersed in the matrix. In the conductive network, numerous electric paths are available through directly-contacted nanoparticles and indirectly-contacted nanoparticles via the quantum tunneling effect, as illustrated schematically in Figure 1. Therefore, the resistance of each conductive path is the sum of intrinsic resistance of nanoparticles (*R_particle_*) and tunneling resistance between neighboring nanoparticles (*R_tunnel_*), defined as,

(1) $$R=R_{particle}+R_{tunnel},$$

where the resistance of nanoparticles themselves can be neglected *(R_particle_* ~ 0) compared with that of the polymer, and the tunneling resistance can be determined by [27]

(2) $$R_{tunnel}=\frac{V}{AJ}=\frac{2h^{2}d}{3Ae^{2}\sqrt{2m\lambda }}\exp\left(\frac{4\pi d}{h}\sqrt{2m\lambda }\right).$$

In the above, *J* denotes the tunneling current density, *V* the electrical potential difference, *e* the quantum of electricity, *m* the mass of electron, *h* the Planck’s constant, *d* the inter-particle distance, *λ* the potential height of insulating layer (0.5–2.5 eV for epoxy), and *A* the cross-sectional area of the tunnel. When the strain is induced at low-level such as UGW traversing, as a result, the piezoresistivity manifested by Nano-NSS can be mainly attributed to triggered tunneling effect by the change in the inter-particle distance since the UGW-induced strain is too small to break up electric paths. Such a change can be described as a function of strain:

(3) $$\frac{\Delta R}{R}=\frac{\Delta R_{tunnel}}{R_{tunnel}}=(1+\varepsilon )\exp\left(\frac{4\pi d_{0}\varepsilon }{h}\sqrt{2m\lambda }\right)-1,$$

where *ε* signifies the coupled strains locally measured by Nano-NSS. If the strain is large enough to break up the linkage among nanoparticles, the resistance changes as a joint consequence of the triggered tunneling effect and breakage of electric paths [28], as

(4) $$\frac{\Delta R}{R}=(1+\varepsilon )\exp\left(\alpha \varepsilon +\beta \varepsilon^{2}+\gamma \varepsilon^{3}+\eta \varepsilon^{4}\right)-1,$$

where *α*, *β*, *γ* and *η* are three constants that can be determined by experiments.

Targeting high sensitivity to UGWs, the supply of nanoparticles connected via the tunneling effect shall be sufficient, in order to ensure the formed Nano-NSS responsive to UGW-induced weak strains (as commented earlier that UGW signals feature ultralow magnitudes), and otherwise the directly contacted nanoparticles will form a stable and saturated conductive network which responds inertly to UGWs. Good dispersion and well controlled nanoparticle content within the percolation region are the key to achieve this, and in such a way the UGW-induced strains can alter the structure of Nano-NSS at a phenomenal degree.

As representative conventional piezoresistive sensors, metal strain gauges, relying on macroscopic mechanical deformation, usually have good performance in measuring static, quasi-static or low-frequency strains. Due to strong directivity of the filaments inside, a metal strain gauge can only capture strain along the filament direction. However, as the frequency of applied strain increases, the material hysteresis becomes a key factor leading to the drop of the gauge factor and an increase of the phase delay [29]. The cutoff frequency is limited by the gauge length which is usually several millimeters. Thus, a 3-mm gauge can remain its stable measurement under the loads of a frequency up to 290 kHz with strain level higher than 300 με [29]. On the other hand, UGWs cause high-frequency multi-direction strains (several hundred kilohertz to megahertz) with ultralow magnitude (<10 με), which are beyond the sensing capability of a metal foil strain gauge. In UGW-based SHM, piezoceramic sensors, optical fibres and laser vibrometers are prevailingly used to capture UGWs.

**Figure 2:**
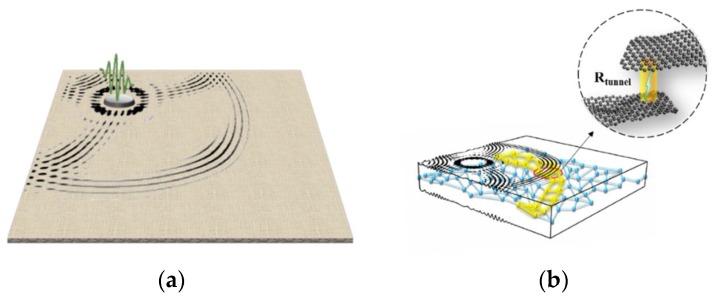
Concept of self-sensing UGWs: (**a**) UGWs, excited by a PZT wafer, propagating in a nano-engineered composite laminate; (**b**) Nano-NSS modulated by UGWs, triggering tunneling effect and leading to local change in electrical resistance.

The innovative piezoresistivity of Nano-NSS in nano-engineered composites makes use of the tunneling effect to perceive UGWs, instead of macroscopic mechanical deformation, endowing the composites with a capability of responding elastic disturbance in broadband without hysteresis, from quasi-static loads, through vibration loads to UGWs, as illustrated in Figure 2.

On top of that, opposing to conventional sensors including metal strain gauges and piezoceramic wafers that are to be glued on the sample surfaces, a Nano-NSS comprises of numerous electric paths which are available through directly-contacted nanoparticles and indirectly-contacted nanoparticles via the quantum tunneling effect that are dispersed in the composites, and thus the acquisition of strains (induced by quasi-static loads, vibration or UGWs) can be performed at any interested areas throughout the entire composites—a key feature that differentiates the Nano-NSS-based SHM from conventional SHM based on the use of external distributed sensors.

### 2.2. Fabrication of Graphene-Nanoparticle-Networked Self-Sensing System (gNano-NSS)

**Figure 3:**
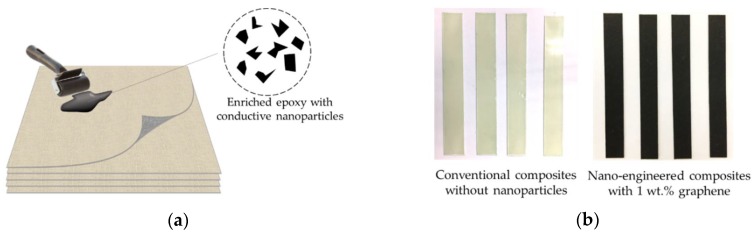
(**a**) Schematic of manufacturing the nano-engineered composite laminate; (**b**) Trimmed samples without/with nanoparticles (1 wt %).

To determine the most suitable candidate nanoparticle and form a Nano-NSS in nano-engineered composites that is responsive to UGW signals faithfully, the electrical properties of three representative types of carbon-based nanoparticles/epoxy hybrid were compared first, namely one-dimensional (1D) rope-like multi-walled carbon nanotube (FloTube^TM^ 7000), two-dimensional (2D) disk-like graphene (Tanfeng Tech GRF-H-FLG-01), and zero-dimensional (0D) sphere-like carbon black (Cabot N220). Epoxy resin (Araldite GY 251) and hardener (Aradur HY 956) were used as the matrix (detailed parameters of nanofillers can be seen in Table A1). The epoxy resin was pre-treated on a hot plate by heating it up to 80 °C for decreasing the viscosity. Mechanical stirring was applied to mix nanoparticles with epoxy resin for 30-min, followed with a sonication in a bath-type sonicator (Branson 2510) for another 5-min. Subsequently, plain-woven glass fibre fabrics (Colan AF 218) were impregnated with the nanoparticle/epoxy hybrid using a hand roller (as in Figure 3a) following a hand layup process, to produce a series of 8-layer laminates. The laminated composites were cured at a room temperature for 24-h using a standard vacuum bagging curing procedure. Such produced laminates (~1.4 mm thick) were trimmed to various sizes of samples. As observed in Figure 3b, distinct from conventional glass fibre/epoxy composite that is semitransparent, such produced composite laminates with nanoparticle/epoxy hybrid manifest opaque, as the matrix was full of dark nanoparticles.

### 2.3. Morphological Investigation

**Figure 4:**
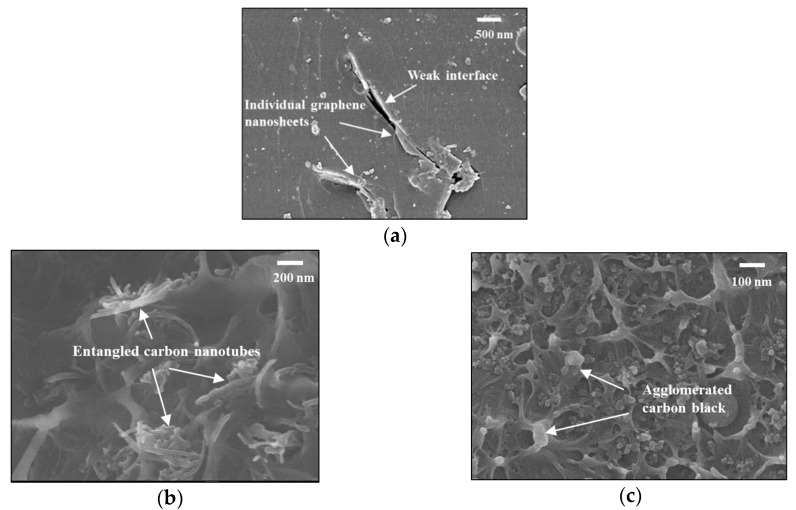
SEM micrographs of the cryo-fractured surfaces of the hybrid with different types of nanoparticles: (**a**) graphene, (**b**) nanotube, (**c**) carbon black.

The morphology of dispersed nanoparticles in nanoparticle/epoxy hybrid was scrutinized with cryo-fractured cross sections of samples using a scanning electron microscopy (SEM) (JEOL Model JSM-6490). SEM images show that the 2D disk-like graphene-based nanoparticles are uniformly apart from each other (Figure 4a), while 1D rope-like carbon nanotube (CNT)-based and 0D sphere-like carbon black (CB)-based nanoparticles are entangled (Figure 4b) or agglomerated (Figure 4c). The macroscopic properties of nanoparticle-enriched composite laminates are substantially influenced by the morphological features of the nanoparticle/epoxy hybrid.

**Figure 5:**
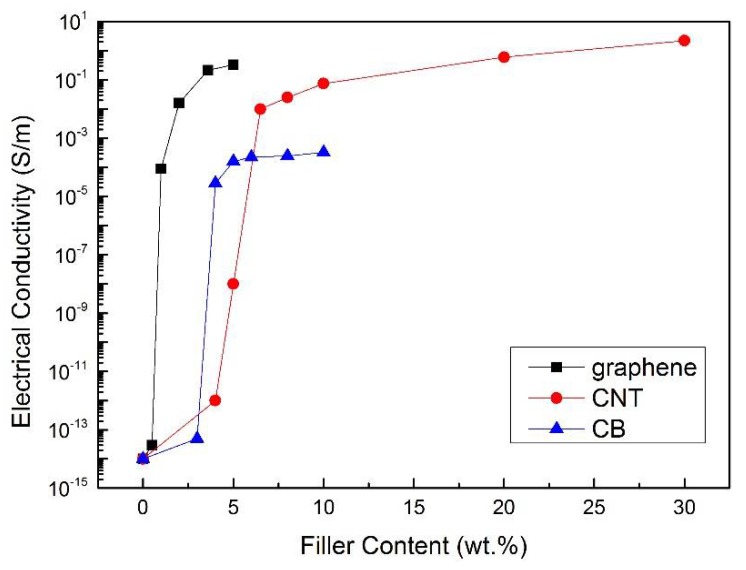
Electrical conductivity of nanoparticle/epoxy hybrid versus weight percentage of nanofiller content.

Samples made from the nanoparticle/epoxy hybrid with different degrees of nanoparticle contents were prepared to examine their electrical properties, on which basis the percolation thresholds of the formed conductive networks in the hybrid were ascertained. According to the percolation theory, the electrical conductivity of the nano-engineered composites has a power-law relationship with the nanofiller content as [30]

(5) $$\sigma \propto {\left(p-p_{c}\right)}^{t},$$

where *σ* signifies the electrical conductivity of the composites, *p* the volume fraction of nanoparticle, *p_c_* the percolation threshold of the composites, and exponential *t* a constant associated with the dimensionality of the conductive nanocomposites. Figure 5 shows the obtained relationship between the electrical conductivity in logarithmic scale and weight percentage of the nanofiller content, from which the percolation thresholds were estimated to be within 0.5–1 wt % for graphene/epoxy hybrid, 4–5 wt % for carbon nanotube/epoxy hybrid, and 4–6.5 wt % for carbon black/epoxy hybrid, respectively. The percolation results are well conformed to results from others’ works, which varied from 0.1 wt % to 8 wt % with different nanofillers [31,32,33,34]. At the threshold, a steep increase in electrical conductivity from nonconductive to conductive region can be observed. As commented earlier, a high sensitivity of Nano-NSS is assured when the nanofiller content is within the percolation region. Upon comparison, it is surmised that the 2D structure of graphene, compared with 1D rope-like carbon nanotube and 0D sphere-like carbon black, facilitates the formation of effective conductive electric paths (via tunneling effect) in the network while avoiding possible agglomeration or entanglement, endowing the hybrid with the lowest percolation threshold. Therefore, 2D disk-like graphene-based nanoparticles were selected as the functional nanofillers to form the dispersed and networked sensing system (gNano-NSS). Based on the morphological analysis and considering the suitable resistivity for further measurement and convenience in fabrication process, 1 wt % (approaching the percolation threshold) graphene was adopted for developing gNano-NSS.

Nevertheless, comparing to the deformation of the polymer matrix, the deformation of conductive nanoparticles within is fairly weak due to their much higher moduli than those of polymers, as well as the poor stress transfer through the interface between nanoparticles and matrix (see Figure 4a). This speculation excludes the possibility that the piezoresistivity manifested by the Nano-NSS under strains could originate from the piezoresistivity of individual nanoparticle [35], rather than inter-nanoparticle change due to matrix deformation.

## 3. Self-Sensing of Quasi-Static Strains

### 3.1. Test Setup

**Figure 6:**
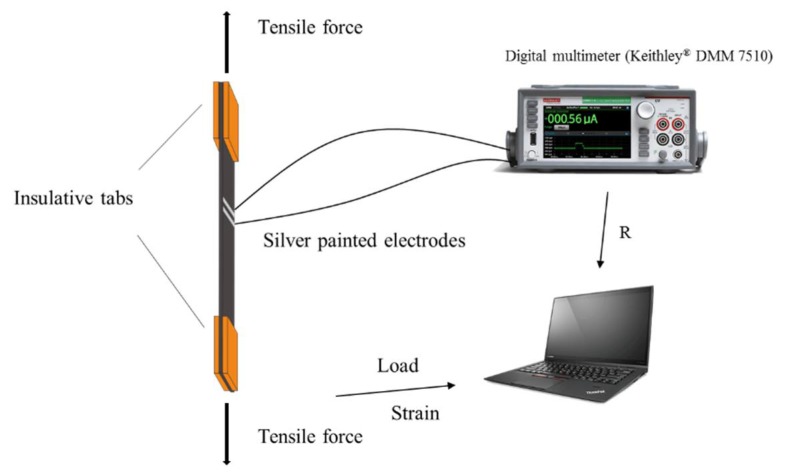
Experimental setup of quasi-static tensile test with simultaneous measurement of resistance change in a local gNano-NSS.

Composite laminates with dispersed gNano-NSS were fabricated, each measuring 1.4 mm thick, 25 mm wide and 200 mm long. Each sample was treated with a 20 mm long insulative tab at the end. Four samples of this kind were tested on a MTS tensile machine (Alliance RT/50) for measurement of mechanical and piezoresistive properties. During measurement, the sensing area on the surface of each sample is the region confined by a pair of silver electrodes with 1 mm gap. The gap was prudently determined and controlled to possess an electrical resistance that is compatible with that of the digital multimeter. Note that according to Equation (7), the static resistance will not influence the resistance changing ratio or sensitivity. The electrodes were connected to a dynamic digital multimeter (Keithley DMM 7510), as shown schematically in Figure 6.

### 3.2. Results and Discussions

**Figure 7:**
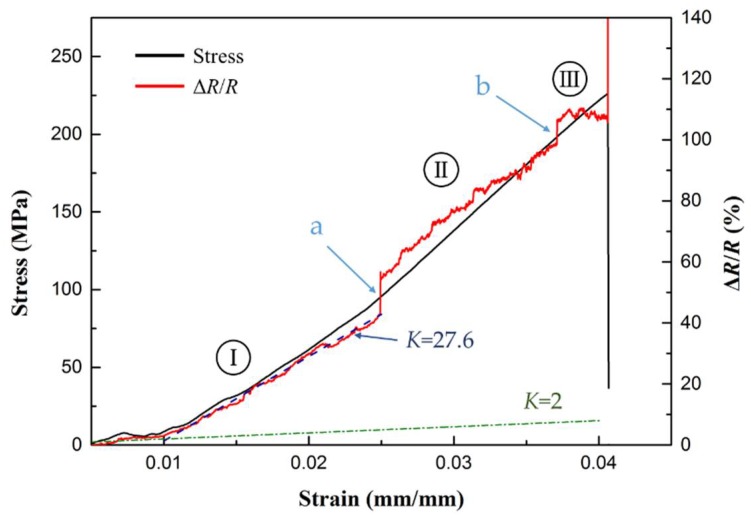
Stress and resistance change ratio of gNano-NSS under quasi-static strain.

Results of the stress/resistance change ratio versus strain applied on the sample are plotted in Figure 7. In general, a failure process of the laminated composites usually initiates from matrix cracking which causes interfacial failure or debonding, followed with fibre breakage or pull-out as stress increases [36]. In Figure 7, dramatic increase in electrical resistance can be clearly observed at point a and b, which partition the failure process into three stages. This step-wise increase in the resistance can be attributed to the occurrence of interphase damage in the composites [37]. Thus the resistance change of gNano-NSS in three stages can be used to identify the failure progressing of nano-engineered composites:

1. the change in resistance experiences a steady increase with the strain (except at the beginning where stress curve is not linear due to the preloading of the machine). No major damage occurs at this stage and the gauge factor (*K*) is calculated based on a linear fitting of the measured strain from 1% to 2.5% (blue dash line in Figure 7) by
  (6) $$K=\frac{\Delta R}{R}/\Delta \varepsilon ,$$
  The obtained gauge factor of the gNano-NSS is ~14 times higher than that of conventional metal strain gauges (*K* = 2, green dash line in Figure 7). According to Equation (6), the gauge factor represents the sensitivity of gNano-NSS through resistance changing ratio with applied strains;
2. when stress level exceeds a certain degree, the damage initiates as small cracks in the matrix. Matrix cracking happens at multiple locations and develops as interface debonding. The first interfacial failure occurring within sensing area is characterized by a sudden jump in resistance at point a. Since small matrix cracks cannot break up the conductive paths, the gNano-NSS is still functional and the resistance grows with similar slope versus strain as before. However, at this stage the damage develops as matrix cracking growth and fibre debonding, which propagates the strain disturbance to the sensing area. Thus the resistance curve fluctuates; and
3. after stress reaches about 85% of the value of failure stress, another jump in the resistance curve appears at point b which corresponds to the begin of fibre cracking, open-up and pull-out. Since every longitudinal fibre goes through the sensing area, a surface-nearby fibre break significantly affects the gNano-NSS at sensing area and causes the sudden increase at point b. The resistance curve at this stage stops regular increase but aggressive fluctuation disrupted by continuous development of severe damage. Until the final material failure with multiple fibres breaking at once, the resulted strong strain release shakes off the cables from electrodes.

**Figure 8:**
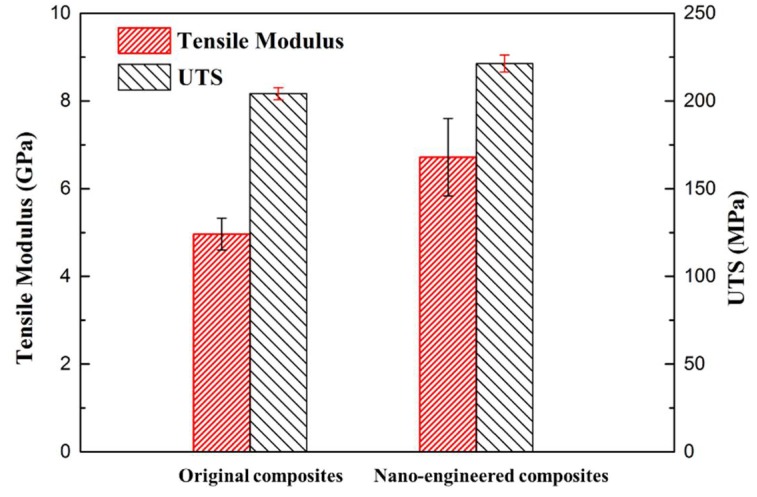
Mechanical properties of the original composite and nano-engineered composite.

Even though only one “dispersed sensor” of the gNano-NSS in the composite was tested, the resistance change can still give rich information about damage occurrence and development not just at sensing area between electrodes but also within the whole structure, unlike the surface-attached strain gauges which only reflect the monitored location strain status. The gNano-NSS features a gauge factor as high as 27.6, compared with a gauge factor of 2 offered by a standard metal strain gauge. Furthermore, the results of mechanical properties exhibit that with inclusion of graphene particles the elastic tensile modulus and ultimate tensile strength (UTS) of composites have been improved 35% and 8%, respectively (see Figure 8). The results are higher than in some previous works [38] (15% and −6%, respectively) mainly due to the exclusion of solvents in dispersion methods, which would degrade the crosslink strength of epoxy resin.

## 4. Self-Sensing of Low-Frequency Dynamic Responses of Structural Vibration

Above experiment calibrated the high sensitivity of gNano-NSS with quasi-static strains as large as 1%. To fully understand the sensing capability of gNano-NSS, the dynamic response of the nano-engineered composites was assessed under vibration loads with dynamic low-frequency and medium-level strains.

### 4.1. Test Setup

**Figure 9:**
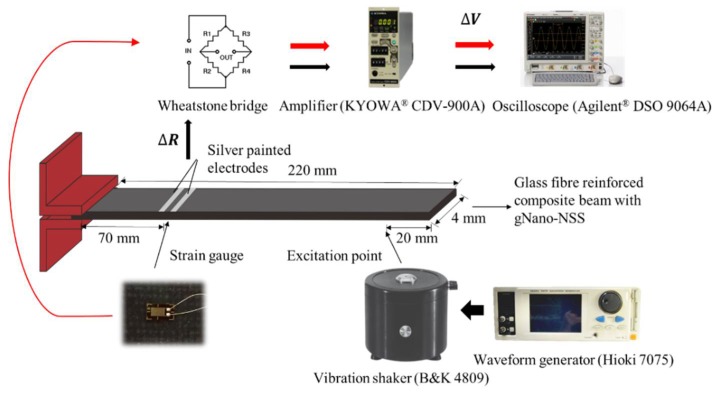
Experimental setup of dynamic vibration test with simultaneous measurement of resistance change in a local gNano-NSS and a counterpart strain gauge.

A cantilever beam made from the above nano-engineered composites, 1.4 mm thick, 220 mm long and 4 mm wide, was fabricated and fixed at one end (Figure 9). An electromagnetic shaker (B&K 4809) was used to introduce a point-like excitation to the beam, 20 mm from the free end. As in the above tensile test, “dispersed” sensing was implemented by painting silver electrodes on the beam surface at 70 mm from the fixed end. The gap was selected and controlled to possess an electrical resistance that is compatible with that of the Wheatstone bridge in the signal amplifier. Since the surface tension and compression strain correlates to the deflection of the monitored beam section, measuring the change in resistance of the local gNano-NSS provides information of the mode shapes under any vibration frequency. For calibration, a conventional metal strain gauge (fence length 5 mm) was pasted on the opposite side of the painted electrodes. During the vibration test, the sensing point and strain gauge were subjected to inverse strain changing, thus the signals captured feature a phase difference of *π*. To capture stable and strong signals under the relatively small strain level of beam subject to vibration loads, a commercial signal amplifier (KYOWA^®^ CDV-900A) with a Wheatstone bridge was used to process signals from both the strain gauge and cable-extracted local gNano-NSS before being outputted to and collected by an oscilloscope (Agilent^®^ DSO 9064A). The sensing performance was examined from 5 Hz to 20 kHz by sinusoidal excitation from a waveform generator (Hioki 7075). The metal strain gauge was used to calibrate the sensing capability of the gNano-NSS with excitation frequency lower than 2 kHz because metal strain gauge failed to respond to dynamic vibration of higher frequency.

### 4.2. Results and Discussions

**Figure 10:**
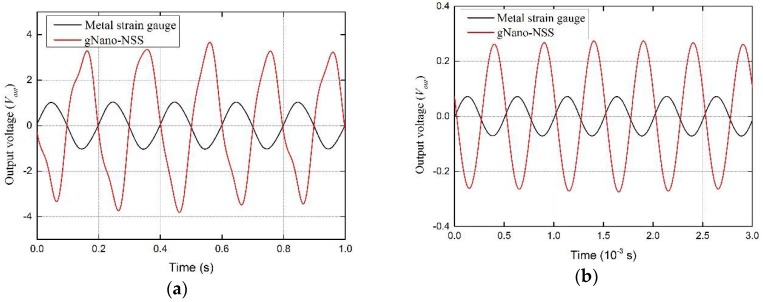
Vibration signals of metal strain gauge and gNano-NSS at (**a**) 5 Hz and (**b**) 2 kHz.

As representative results, the obtained vibration signals of strain gauge and gNano-NSS at 5 Hz and 2 kHz are plotted in Figure 10. The strain values at the monitored beam point can be ascertained according to the configuration and specification of the signal amplifier and the Wheatstone bridge [39]:

(7) $$\varepsilon =\frac{4V_{out}}{GV_{bridge}\cdot K},$$

where *G* represents the internal gain of the amplifier (the maximum ×10,000 used here for both strain gauge and gNano-NSS), *V_out_* the output voltage signals, *V_bridge_* the excitation voltage of Wheatstone bridge (2 V used here) and *K* is the gauge factor of the sensor. With the known gauge factor of the metal strain sensor (*K* = 2) and the output voltage signals of metal strain gauge and the gNano-NSS, the *K* of the self-sensing capability was calculated, which is about 7.2 at excitation of 5 Hz and 7.59 at excitation of 2 kHz. The slight difference under different frequency can be attributed to the different levels of strain. The gauge factor under vibration loads (measured strain ±20 με~±400 με (±0.002%~±0.04%)) is fairly lower than that measured under tensile loads (strain 1%~2.5%). This observation has verified that the sensing mechanism features a complicated trend: at low-level strain, tunneling effect is the dominating factor while at high-level strain the resistivity increases more rapidly due to the break-up of electric paths. The response signals of other frequencies are illustrated in Figures S1 and S2, from which it is clear to see no obvious hysteresis observed in the response signals of gNano-NSS in a wide bandwidth.

## 5. Dispersed Self-Sensing of UGWs

The nano-engineered composites have already been demonstrated effective and accurate in acquiring quasi-static loading with high-level strain and dynamic loading with medium-level strain. To further study the possibility to combine novel nano-engineered composites with UGW-based SHM, the self-sensing capability of gNano-NSS in perceiving UGWs with high frequency and low-level strain was examined.

### 5.1. System Setup

**Figure 11:**
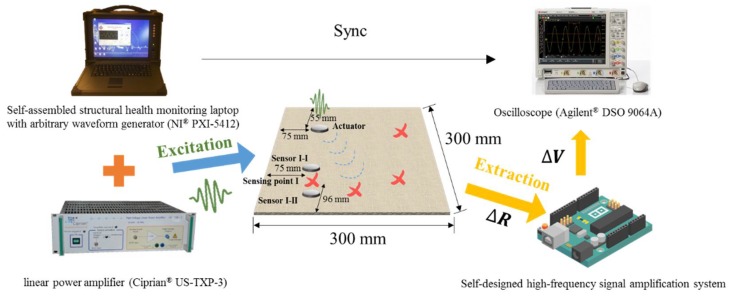
Experimental setup of the excitation and extraction system in the acquisition of UGW test.

A 300 mm × 300 mm 8-layer composite laminate plate (1.4 mm thick) with the gNano-NSS was fabricated to test the capability of sensing ultrasonic waves. A piezoelectric lead zirconate titanate (PZT) actuator (Haiying Enterprise Group Co., Ltd., Wuxi, China, P-51, 12 mm in diameter, 1 mm in thickness) was mounted on the surface of the laminate to excite probing ultrasonic signals with a self-developed SHM system. The system consists of UGW generation module comprising of an NI PXI-5412 arbitrary waveform generator (AWG) and a linear power amplifier (Ciprian^®^ US-TXP-3). The system generates tailor-made probing guided waves in narrow-band waveforms at an arbitrary frequency in the range of 0–2.5 MHz. The AWG-generated probing UGW signals were amplified by the linear power amplifier before being applied on the actuator. As illustrated in Figure 11, multiple locations of the structure were arbitrarily selected as “dispersed sensors” to measure the resistance change with ultrasonic wave propagating through the samples. To obtain strong and stable signals, a dedicated high-frequency signal amplification system was designed and developed, comprising of an electronic amplifier circuit, and a series of filters. The Wheatstone bridge was rebuilt with an adjustable balance resistor which greatly facilitated the electrodes painting process, unlike the fixed 120 Ω and 350 Ω in commercial bridges that require careful gap control of electrodes. The change in local electrical resistivity was then transferred into voltage signals and collected via an oscilloscope (Agilent^®^ DSO 9064A). To verify the sensing performance, the excitation UGW signals were varied from 50 kHz to 440 kHz and captured signals were compared with PZT sensors attached nearby, for example sensor I-I and I-II around sensing point I in Figure 11.

### 5.2. Results and Discussions

**Figure 12:**
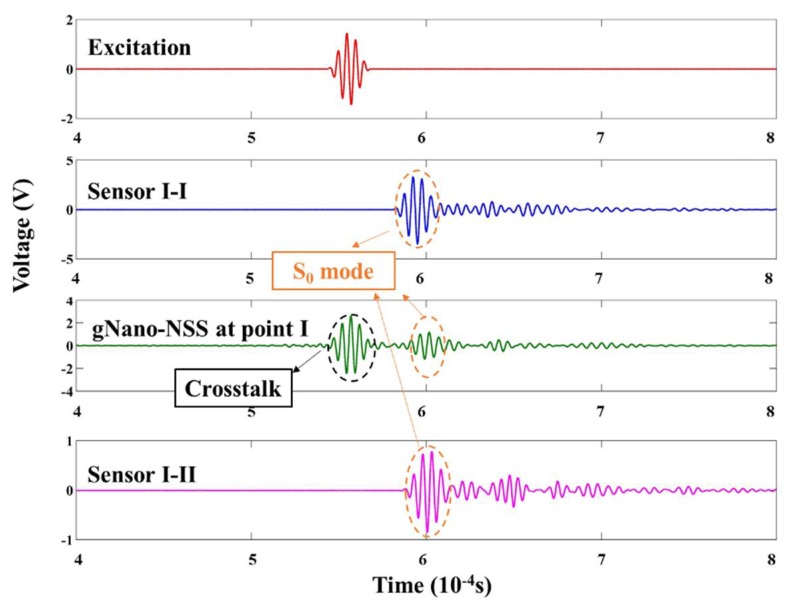
Raw UGW signals captured by the gNano-NSS of the composite and counterpart PZT sensors at point I.

**Figure 13:**
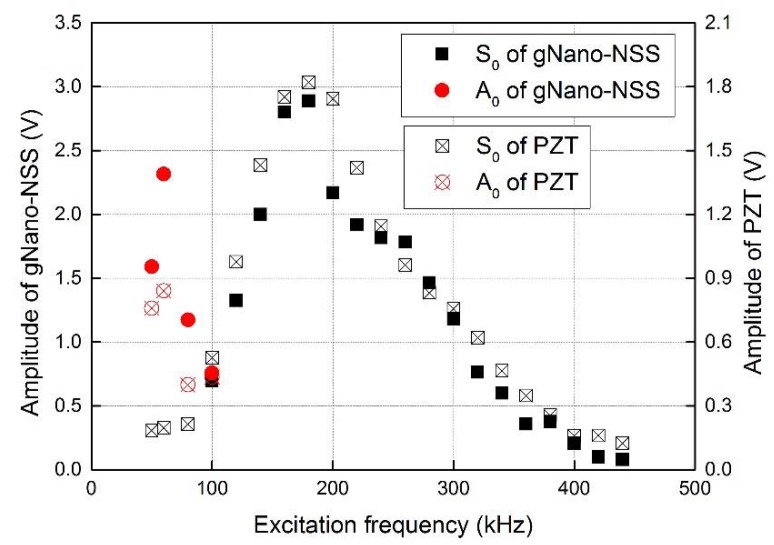
Comparison of time-frequency-amplitude response signals of UGW propagation in laminates captured by the gNano-NSS and by PZT sensors at point I.

As some representative results, the captured signals of gNano-NSS at point I with excitation frequency of 180 kHz is demonstrated in the time domain in Figure 12. It can be clearly observed that the first-arrival wave component (viz., the zeroth-order symmetric Lamb wave mode guided by the laminate, denoted by S_0_ hereinafter) of the local gNano-NSS has good agreement with the signals of PZT sensors in term of the arrival time. The obvious extra part of the acquired signal is the crosstalk noise at the initial moment of excitation which comes from the high voltage signal generation system. Figure 13 shows the amplitude change with the frequency. The captured signals of the gNano-NSS have similar trend with conventional PZT sensors in both S_0_ and A_0_ (The zeroth-order anti-symmetric Lamb wave mode, denoted by A_0_ hereinafter) modes, although with a slight difference in the magnitude due to the different couple effect in sensing mechanisms discussed earlier. However, because the crosstalk noise is broad enough to cover nearby signals at low frequency, some data points of S_0_ wave are missing in Figure 13.

This experiment has proven that the gNano-NSS has the capability of sensing the ultrasonic waves with little delay, high signal-noise ratio and comparable performance with conventional PZT sensors. Since the gNano-NSS is fully integrated with the composites, the function as “dispersed sensors” can be performed at any desired positions.

## 6. Concluding Remarks

Well-dispersed Nano-NSS endows conventional composites with a versatile sensing capability of a broad type of loads. The sensing function is realized as “dispersed sensors” and combined with well-established SHM techniques to develop new way of damage sensing and monitoring. In this study, self-sensing functional nano-engineered composites were developed with gNano-NSS. Experiments were elaborately designed to calibrate and verify their sensing performance. In quasi-static test, local resistance change measurement of gNano-NSS has shown sensitive relationship with the strain of the monitored area, which could provide more information of damage status within materials than merely surface strain response from conventional attached sensors. The observed high sensitivity was also found in the vibration test, during which gNano-NSS showed no obvious delay and deviance in low-frequency dynamic response. The acquisition of UGW test promoted the sensing capability to the ultrasonic regime and low-level strains. The gNano-NSS exhibits fast, stable and accurate response to broadband dynamic strains yet less intrusion to the original composites, which pioneers new sensing techniques in developing structural health monitoring systems. As an additional merit, mechanical profiles of the composites have also been improved, benefiting from the networked graphene nanoparticles in polymers. To take a step further, the wires and cables used in the sensing network can further be eliminated using techniques such as screen-printed or 3D-printed circuits, to endow the structures with a fully integrated SHM capability. The application of gNano-NSS in damage localization with UGW-based SHM will be presented in future work.

## Supplementary Materials

The following are available online at http://www.mdpi.com/1424-8220/18/5/1398/s1, Figure S1: Vibration responses measured under frequencies of 100, 200, 500 and 1000 Hz. The amplitudes of the signals have been adjusted to clearly show the waveforms identified; Figure S2: Vibration responses measured under frequencies of 5, 10 and 20 kHz. The amplitudes of the signals have been adjusted to clearly show the waveforms identified.

## Appendix A

**Table A1:** Detailed parameters of nanofillers.

Nanofiller	Particle Dimensions	
Carbon nanotube	~6–10 nm (tube diameter)	~50 μm (length)
Graphene	~30 μm (lateral diameter)	~3 nm (thickness)
Carbon black	~80 nm (diameter)	-
