# Supplementary figures and images for: Dispersed Sensing Networks in Nano-Engineered Polymer Composites: From Static Strain Measurement to Ultrasonic Wave Acquisition
Source: Sensors (Basel). 2018 May 2;18(5):1398. doi: 10.3390/s18051398 (PMC5982692; doi:10.3390/s18051398)

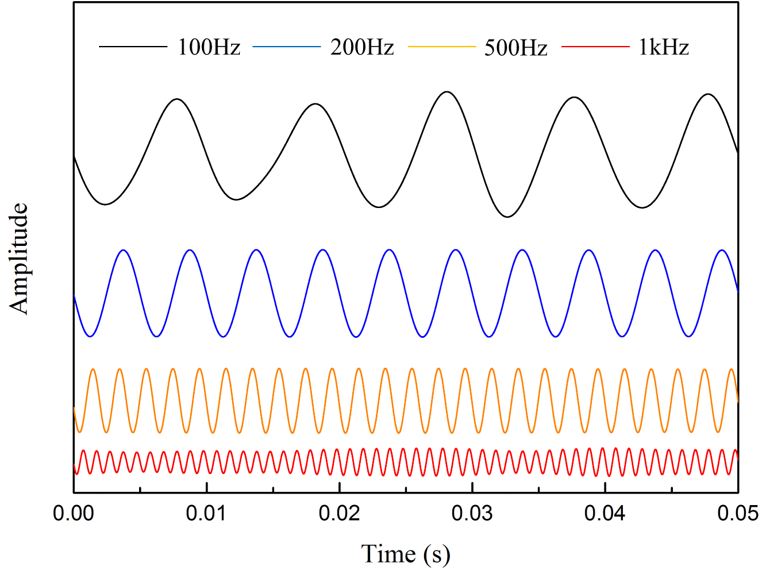

Supplement: Supplementary file 1 [file sensors-18-01398-s001.zip › sensors-288412-SI/Figure S1.tif]

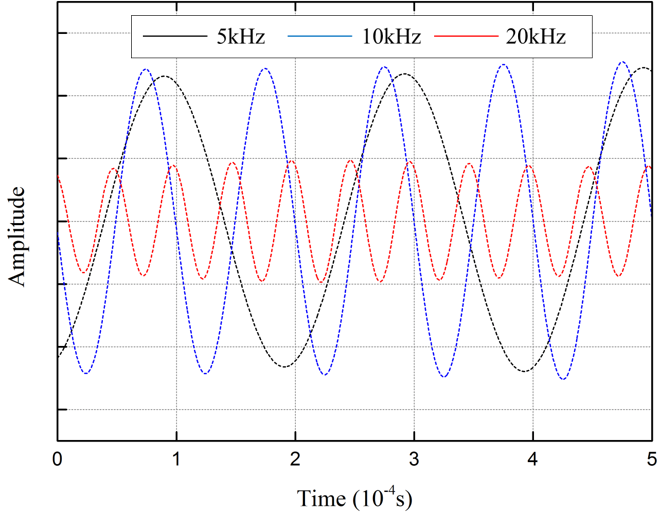

Supplement: Supplementary file 1 [file sensors-18-01398-s001.zip › sensors-288412-SI/Figure S2.tif]
